# CD11c/CD18 Dominates Adhesion of Human Monocytes, Macrophages and Dendritic Cells over CD11b/CD18

**DOI:** 10.1371/journal.pone.0163120

**Published:** 2016-09-22

**Authors:** Noémi Sándor, Szilvia Lukácsi, Rita Ungai-Salánki, Norbert Orgován, Bálint Szabó, Róbert Horváth, Anna Erdei, Zsuzsa Bajtay

**Affiliations:** 1 MTA-ELTE Immunology Research Group, Hungarian Academy of Sciences, Budapest, Hungary; 2 Department of Immunology, Institute of Biology, Faculty of Science, Eötvös Loránd University, Budapest, Hungary; 3 Department of Biological Physics, Institute of Physics, Faculty of Science, Eötvös Loránd University, Budapest, Hungary; 4 Nanobiosensorics “Lendület” Group, Institute of Technical Physics and Material Sciences, Centre for Energy Research, Hungarian Academy of Sciences, Budapest, Hungary; Singapore Immunology Network, SINGAPORE

## Abstract

Complement receptors CR3 (CD11b/CD18) and CR4 (CD11c/CD18) belong to the family of beta2 integrins and are expressed mainly by myeloid cell types in humans. Previously, we proved that CR3 rather than CR4 plays a key role in phagocytosis. Here we analysed how CD11b and CD11c participate in cell adhesion to fibrinogen, a common ligand of CR3 and CR4, employing human monocytes, monocyte-derived macrophages (MDMs) and monocyte-derived dendritic cells (MDDCs) highly expressing CD11b as well as CD11c. We determined the exact numbers of CD11b and CD11c on these cell types by a bead-based technique, and found that the ratio of CD11b/CD11c is 1.2 for MDDCs, 1.7 for MDMs and 7.1 for monocytes, suggesting that the function of CD11c is preponderant in MDDCs and less pronounced in monocytes. Applying state-of-the-art biophysical techniques, we proved that cellular adherence to fibrinogen is dominated by CD11c. Furthermore, we found that blocking CD11b significantly enhances the attachment of MDDCs and MDMs to fibrinogen, demonstrating a competition between CD11b and CD11c for this ligand. On the basis of the cell surface receptor numbers and the measured adhesion strength we set up a model, which explains the different behavior of the three cell types.

## Introduction

Monocytes, macrophages and dendritic cells are phagocytes, which are able to adhere to extracellular matrix components (e.g. fibrinogen) via different integrin molecules. Integrins are heterodimeric transmembrane glycoproteins consisting of a non-covalently coupled alpha and beta chain [1]. These molecules mediate several functions that are associated with cytoskeleton rearrangements, including cell-to-cell and cell-ECM contacts, proliferation, phagocytosis and transendothelial migration of immune cells [1–4]. The most abundant integrins expressed by cells of the monocytic linage are complement receptors (CR) CR3 (CD11b/CD18) and CR4 (CD11c/CD18), which are members of the β_2_ integrin family. The main natural ligand of CR3 and CR4 is iC3b, the inactivated fragment of C3, the central complement component [5], however, they bind several other molecules in common, like fibrinogen, ICAM-1, factor X, etc. [6–11].

In humans, CR3 and CR4 are simultaneously expressed in monocytes, macrophages, dendritic cells, neutrophil granulocytes (PMNs) and NK cells. Since the main ligand of CR3 and CR4 is identical, the study of the individual function of these integrins is challenging. In contrast to the human system, murine CD11c/CD18 expression is mainly limited to dendritic cells, therefore CR4 can be used to identify this cell population. Furthermore the function as well as signal transduction mediated by mouse CR3 can be separately studied [12–15]. Results obtained in studies on mouse CD11b/CD18 however cannot be simply translated to the human system, due to the previously mentioned differences between the two species. Our goal is to dissect and determine the individual functional properties of human CR3 (CD11b/CD18) and CR4 (CD11c/CD18).

Earlier we demonstrated that CR3 plays a key role in the phagocytosis of iC3b-opsonized microbes by human MDDCs, while their maturation and inflammatory cytokine production is not influenced by iC3b or CD11b specific antibody [16, 17]. We also examined the role of CD11c/CD18 in the complement mediated phagocytosis of MDDCs, and found it dispensable in this process, proving that the function of CR3 and CR4 is not identical. The aim of the present work is to determine the participation of CR3 and CR4 in a different function linked to β2 integrins; namely cellular adhesion. The absolute numbers and the conformational state of CR3 and CR4 expressed by the cells were assessed and the adherence of normal human monocytes, MDDCs and MDMs to fibrinogen was investigated. The strength and the kinetics of adherence were estimated using classical and state-of-the-art biophysical methods. Our results provide further evidence that human CR3 and CR4 are involved in different cellular functions—despite their capacity to bind the same ligands.

## Results

### Absolute number and conformation analysis of CD11b/CD18 and CD11c/CD18 on monocytes, MDMs and MDDCs

**Fig 1:**
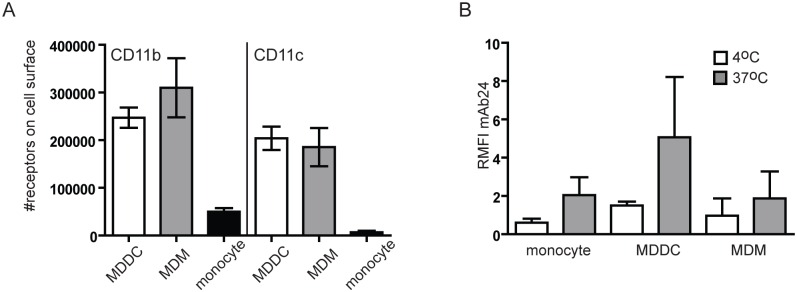
Expression and conformation of CD11b and CD11c. **(A)** The exact amount of CD11b and CD11c on the surface of monocyte-derived dendritic cells (MDDC), monocyte-derived macrophages (MDM) and monocytes were determined using Qifikit (Dako) as described in Materials and methods. Data presented are mean +/-SD of three independent donors’ results. **(B)** Cells were stained with monoclonal antibody mAb24 that is specific for the high affinity conformation of CD18. Relative mean fluorescence intensity (RMFI) was calculated in each case by comparing the signal of mAb24 stained cells to isotype matched control antibody stained cells (RMFI = MFI mAb24/MFI isotype control). At 4°C RMFI values were around 1 (monocytes: 0,60+/-0,22; MDDC: 1,50+/-0,20; MDM: 0,97+/-0,91), meaning that cells do not have active β2 integrins on their surface. At 37°C all cell types bound mAb24 (RMFI for monocytes: 2,05+/-0,93; MDDC: 5,07+/-3,15; MDM: 1,87+/-1,42) showing that β2 integrins were in a conformation capable of ligand binding on their surface. Data presented are mean +/-SD of three independent donors’ results.

Our aim was to compare the role of CD11b and CD11c in adhesion to fibrinogen on monocytes, MDMs and MDDCs. To achieve this goal, first we had to assess the exact number of the receptors expressed by the cells, which has not been determined in a comprehensive manner so far. To this end we used Qifikit (Dako), a bead based flow cytometric technique, which enables the determination of absolute receptor numbers. Although monocytes, macrophages and dendritic cells are thought to express CD11b and CD11c in similarly high amounts, precise numbers assessed by us show significant differences. The number of CD11b molecules on the cell surface is 247174+/-21281 for MDDCs, 309753+/-62045 for MDMs and 49831+/-7810 for CD14+ monocytes. Assessing CD11c expression we detected 203996+/-24623 for MDDCs, 185357+/-40160 for MDMs and 6972+/-2972 for CD14+ monocytes (Fig 1A). We also calculated the CD11b:CD11c ratio on these cell types, and found 1,2 for MDDCs 1,7 for MDMs and 7,1 for monocytes. Namely, the relative amount of CD11c to CD11b is the highest in the case of MDDCs intermediate on MDMs and the lowest on monocytes. This suggests that the functions mediated by CD11c are the most instrumental in the case of MDDCs.

Since ligand binding by integrins is under conformational regulation, and only the open form is known to be active [18], next we examined the conformational state of the receptors. Monocytes, MDDCs and MDMs were stained at 4°C or 37°C with mAb24 that recognizes the active conformation of CD18, the common β2 chain in CR3 and CR4. We found that all cell types are able to bind mAb24 at 37°C, and to a smaller extent at 4°C. The difference was statistically not significant in neither of the cases when analysed by paired t-test. Nevertheless these data clearly show that CR3 and CR4 are in a conformation capable of ligand binding on the surface of monocytes, MDDCs and MDMs (Fig 1B).

### Analysis of adhesion to fibrinogen using classical methods

**Fig 2:**
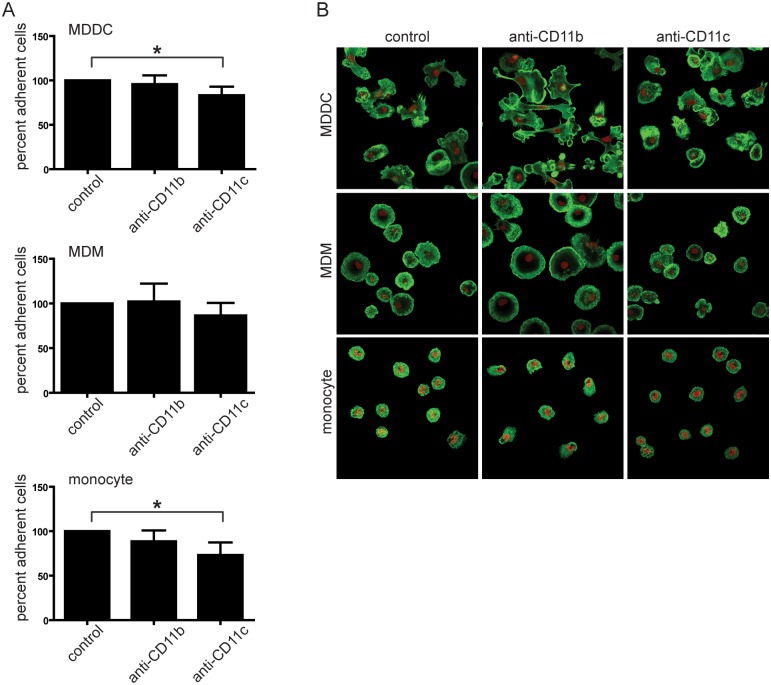
Number and contact zone structure of cells after blocking CD11b or CD11c with antibodies. Cells were treated with monoclonal anti-CD11b or anti-CD11c antibodies on ice for 30 min or left untreated for control. The Fc receptor blocking reagent was used prior adding the antibodies in all samples. Cells were let to adhere for 30min at 37°C 5%CO_2_ on plates coated previously with 10μg/ml fibrinogen and blocked with PLL-g-PEG. After that cells were fixed with 2% paraformaldehyde for 10min, and washed twice with PBS to remove unbound cells. Nuclei were stained with Draq5 and actin cytoskeleton with phalloidin-Alexa488 probe. (**A**) The number of adhered cells was determined by analysing 12 representative fields after each treatment using Olympus IX81 microscope at 10x magnification. The number of adherent cells in control samples was taken 100%, and the effect of different treatments was compared to it. Mean +/- SD of three independent donors’ results is shown. Repeated measures ANOVA with Bonferroni post-test was used to determine significant differences compared to control * = p<0,05 (**B**) A 0,42 μm slice of the contact zone was analysed by 60x magnification. Red fluorescence shows cell nuclei (Draq5), green shows filamentous actin (phalloidine-Alexa488).

To study the individual role of CD11b/CD18 and CD11c/CD18 in the adhesion to fibrinogen we blocked either CD11b or CD11c by ligand binding site specific monoclonal antibodies. Unspecific binding of the antibodies was prevented by adding FcR blocking reagent to all the samples (including controls), and the number of adhered cells was determined as percentage of untreated control samples. Furthermore, treatment with isotype matched control mAbs did not interfere with the adhesion of each cell type to fibrinogen. As shown in Fig 2A, blocking CD11c decreased the number of adhering MDDCs and monocytes significantly, and slightly (not significantly) blocked the adherence of MDMs. Blocking CD11b had no effect on MDMs and MDDCs, however decreased the number of adhered monocytes slightly (not significantly).

Next we aimed to determine how blocking of CD11b/CD18 or CD11c/CD18 receptors affects the properties of adhesion to fibrinogen. To this end we analysed the contact area of the differently treated MDMs, MDDCs and monocytes by confocal microscopy. Actin cytoskeleton and nuclei were stained, and 0,42 μm optical sections of the contact zone were analysed. Fig 2B shows that blocking CD11b on MDMs results in larger contact areas, while blocking CD11c decreases them compared to untreated samples. In the case of MDDCs inhibition of CD11b induced a more polarized and slightly larger contact surface, while anti-CD11c treated cells showed a round shape, but similar contact area to control cells. Monocytes are smaller than MDMs and MDDCs, therefore their contact zone is also smaller, as seen in Fig 2B. Blocking of CD11b caused an increased contact area also in the case of monocytes, in contrast to inhibition of CD11c, which had no effect.

**Fig 3:**
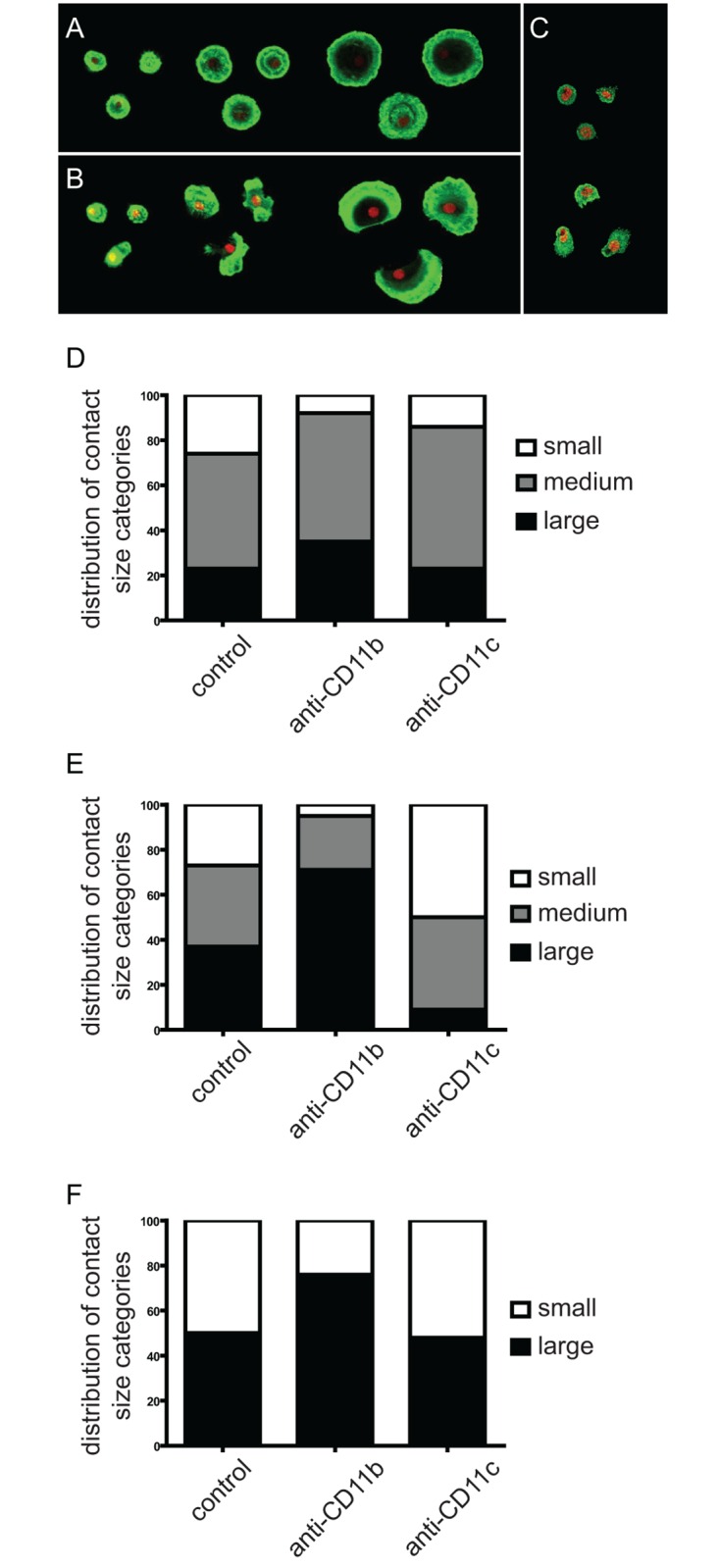
Contact zone size of cells after blocking CD11b or CD11c with antibodies. Cells were divided into categories based on the size of their contact zones. MDMs (**A**) and MDDCs (**B**) were categorised into the following three groups: small ≤2000 pixel^2^, medium 2000–400 pixel^2^, large ≥4000 pixel^2^. For monocytes (**c**) only 2 categories were established because of their smaller size (small ≤1000 pixel^2^, large> 100 pixel^2^). 200 cells were counted for each cell type and each treatment, and the distribution between the different contact size categories was determined and is shown for MDDCs (**D**), MDMs (**E**) and monocytes (**F**). Results of one representative experiment of three independent ones is shown.

To quantify these observations, we established different categories based on the contact size of the cells. Namely we defined 3 categories for MDMs and MDDCs and 2 for monocytes (Fig 3A–3C), and determined their proportion. By blocking CD11b, cells with small contact area almost completely disappeared in the case of MDMs and MDDCs, and their proportion decreased in the sample of monocytes. Simultaneously, the ratio of spread-out cells increased in the case of all cell types, showing that CD11b acts against spreading. On the contrary, blocking CD11c elevated the ratio of cells with small contact area in MDMs and the ratio of medium area cells in MDDCs, but had no effect on monocytes (Fig 3D–3F).

### Analysis of adhesion force using computer controlled micropipette

**Fig 4:**
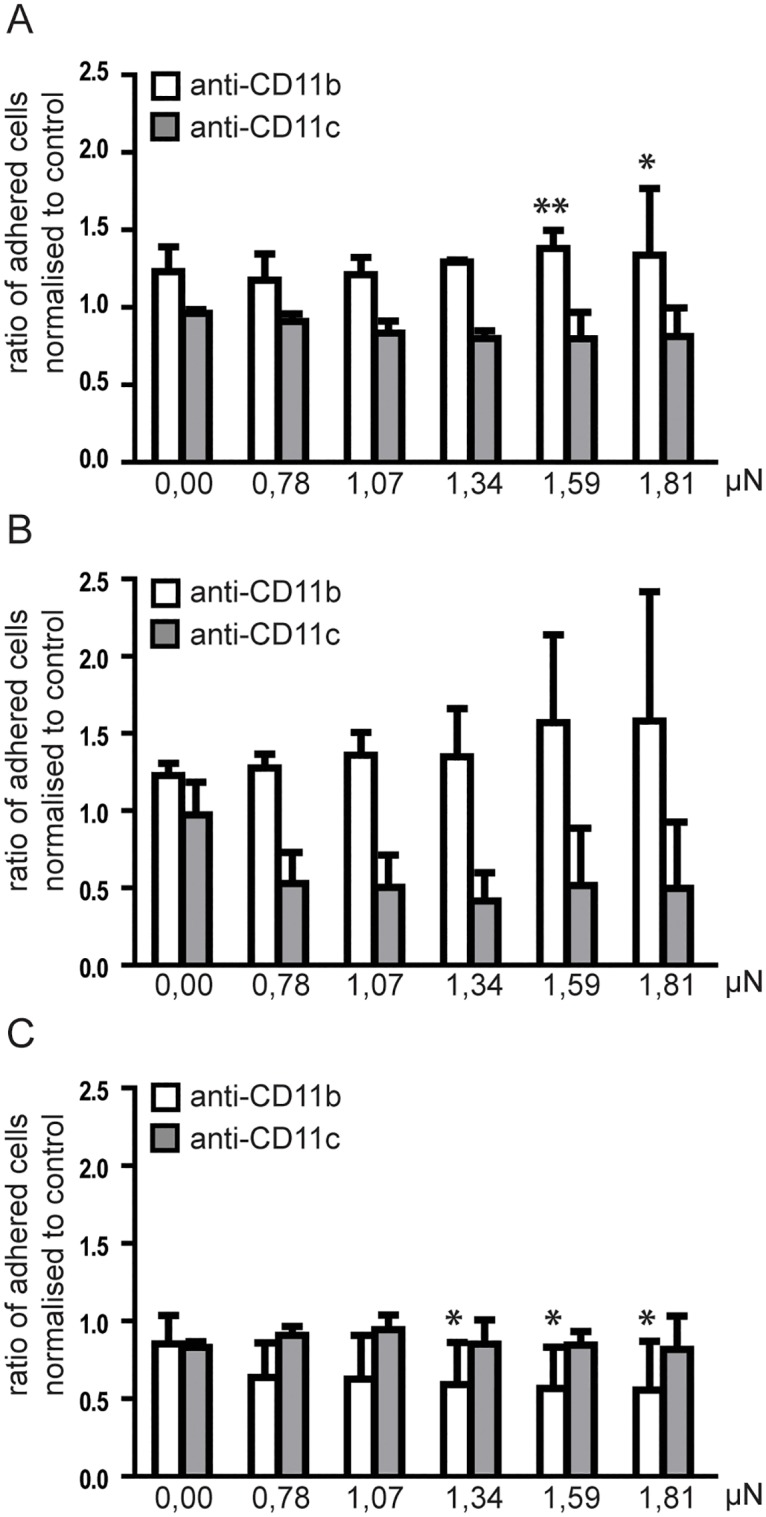
Force of cell adhesion after blocking CD11b or CD11c with antibodies. MDDCs (**A**), MDMs (**B**) and monocytes (**C**) were treated with monoclonal anti-CD11b or anti-CD11c antibodies on ice for 30 min or left untreated for control. The Fc receptor blocking agent was used prior adding the antibodies in all samples. Cells were let to adhere for 30min at 37°C 5%CO_2 i_n Petri dishes coated previously with 10μg/ml fibrinogen and blocked with PLL-g-PEG. After that cells were gently washed twice with PBS to remove unbound cells. The number of adhered cells was determined in the field of the microscope and is shown as 0,00μN. The computer controlled micropipette made serial pick-up processes in the field by using increasing amount of vacuum. A microscopic picture was taken after each round and the number of remaining cells was determined. The ratio of adhered cells was determined at each lifting force value by dividing the number of adhered cells in the anti-CD11b or anti-CD11c blocked samples by the number of cells in corresponding control sample. Data presented are mean +/-SD of three independent donors’ samples. Repeated measures ANOVA with Bonferroni post-test was used to determine significant differences compared to control at each force. * = p<0,05, ** = p<0,01.

To further characterise the role of CD11b/CD18 and CD11c/CD18 in cell adhesion to fibrinogen, we performed state-of-the-art biophysical measurements on differently treated cells. Cells were let to adhere on fibrinogen coat, and their adhesion force was assessed by trying to pick them up with a computer controlled micropipette using vacuum assisted fluid flow. The pick-up process was repeated several times with increasing the vacuum, and cells remaining on the surface were counted after each cycle. Applied vacuum was converted to force (μN) on the basis of computer simulations, and experimental data are presented as the ratio of differently treated adherent cells compared to the untreated control. Using this method we observed that blocking CD11b increased the force of adhesion of MDDCs significantly and elevated the force of adhesion in the case of MDMs (not significant, Fig 4A and 4B), however, it significantly decreased the strength of adhesion in the case of monocytes (Fig 4C). The significant strengthening and decreasing effect was observed among the cells that adhered the strongest. This is in good concordance with our previous results showing that anti-CD11b treatment slightly reduces the number of adhesive monocytes (Fig 2A). This treatment also increased the proportion of cells with medium and large contact area (Fig 3). Blocking CD11c decreased the adhesion force in each cell type, underlining the importance of this receptor in the process, however the differences were not found to be significant.

### Analysis of adhesion kinetics using optical waveguide biosensor

**Fig 5:**
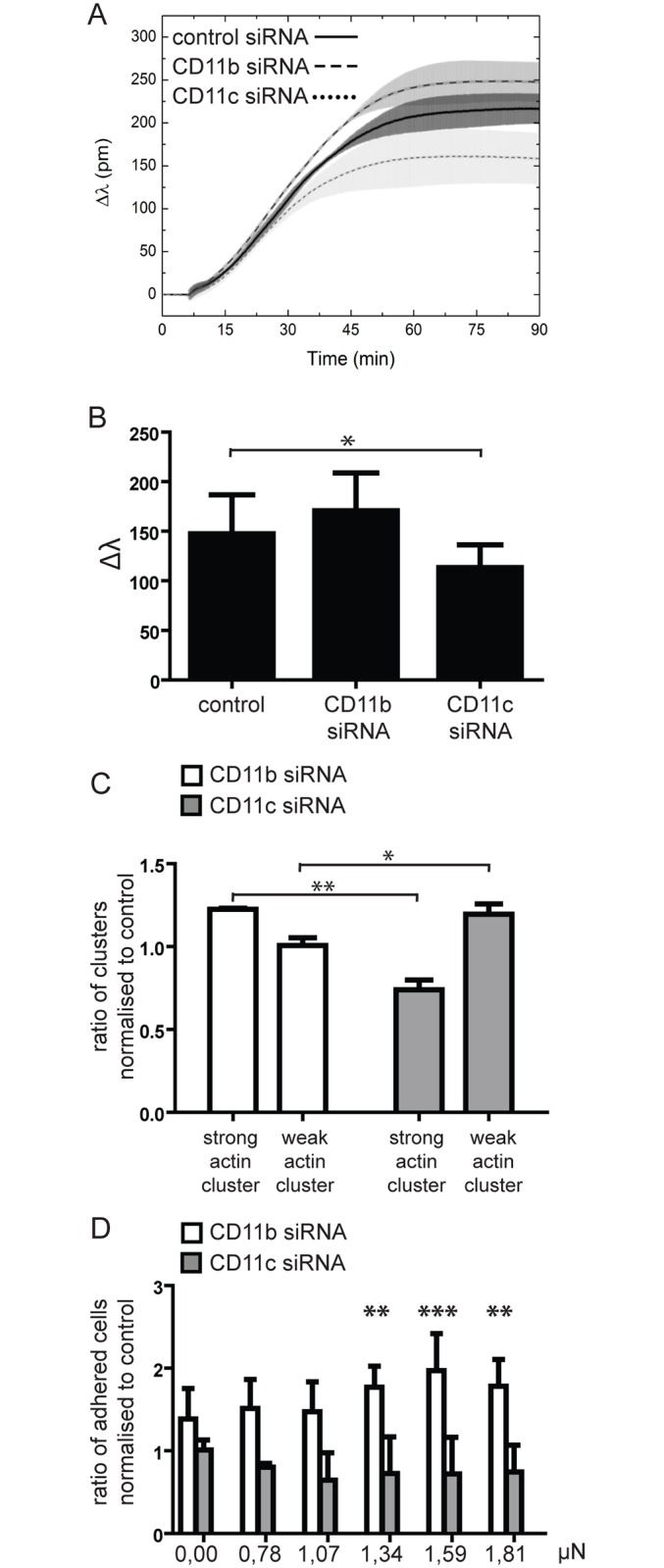
Adhesion of RNA silenced macrophages. MDMs were differentiated under conditions where CD11b or CD11c expression was downregulated by receptor specific siRNA. Control cells were treated with negative control siRNA. (**A**) Kinetic curves of adhering cells was recorded by EPIC BT measurement. Change in refractive index and thereby detected wavelength (Δλ) is plotted against time in the case of CD11b (dashed line), CD11c (dotted line) or negative control (black line) silenced MDMs of the same donor. The shaded area around each line shows the deviation between the parallel samples. One representative measurement out of three independent is shown. (**B**) Average+/-SD Δλ of three independent measurements was determined at the 30^th^ minute of analysis. Paired t-test was used to compare the effect of CD11b or CD11c silencing compared to control siRNA treated samples. CD11c silencing was found to decrease Δλ significantly (p<0,5) (**C**) Cells were let to adhere for 30min at 37°C 5%CO_2_ on plates coated previously with 10μg/ml fibrinogen and blocked with PLL-g-PEG. Filamentous actin was stained with phalloidine-Alexa488 and contact zones were scanned for 200 cells with Olympus IX81 confocal microscope using 60x objective. Pictures were analysed for the amount of strong and weak actin clusters with ImageJ. CD11b and CD11c silenced cells were compared to negative control siRNA treated cells. MDMs with reduced CD11b had significantly more strong clusters and significantly less weak clusters compared to CD11c silenced cells. Results shown are mean +/- SD of three independent experiments, repeated measures ANOVA with Bonferroni post-test was used, * = p<0,05, ** = p<0,01. (**D**) The number of cells adhering with a given force was determined by the computer controlled micropipette. MDMs with silenced CD11b had significantly more cells that adhered with strong force compared to negative control siRNA treated cells. Differences in the case of CD11c silencing were not significant. Results shown are mean +/- SD of three independent experiments, repeated measures ANOVA with Bonferroni post-test was used, ** = p<0,01, *** = p<0,001.

To perform kinetic studies on adherence to fibrinogen, the EPIC label free optical biosensor was used. This method enables the real-time monitoring of a 100–200 nm width layer over the adhesive surface by analysing the refractive index alterations in this volume. Cells can reach this area only by adhesion, thereby non adhering cells are excluded from the measurement. Signal is detected as the shift of resonant wavelength (Δλ). The higher this shift, the larger area of the sensor is covered or the stronger the contact between the cells and their substrate. This means that using this method we detect a combined signal of the number of the adhered cells and the size and density of their contact area [19, 20]. The experiment was performed on MDMs, where the expression of CD11b or CD11c was downregulated using RNA silencing. For control, cells were transfected with negative control siRNA. To avoid the undesired contribution of unbound antibodies to the optical sensor antibody blocking was not used in this method. Fig 5A shows a representative graph, where CD11b silenced cells show higher, and CD11c silenced cells lower signal, as compared to the control sample. Since cells were let to adhere for 30 minutes in the case of the other methods used by us, we determined the mean Δλ value for 3 independent experiments at the 30^th^ minute of the kinetic experiment. Data obtained confirm our previous findings, namely, that blocking CD11c decreased the adhesive capacity of the cells significantly while blocking CD11b elevated it slightly (not significantly) (Fig 5B). To validate the results obtained with RNA silenced cells, we assessed their adhesion profile employing two further methods, too. We analysed the actin clusters of the contact zone in confocal microscope, and found that CD11b silenced MDMs had stronger actin clusters than the control cells, and significantly more than the CD11c silenced cells had. The opposite was true for the weak actin clusters; CD11c silenced cells had significantly more of that than the CD11b silenced cells (Fig 5C). The computer controlled micropipette confirmed these results (Fig 5D) showing that RNA silencing caused similar changes in the cells’ adhesive capacity as receptor blocking with antibodies.

## Discussion

The family of β2 integrins consists of four members: CD11a/CD18 (LFA-1), CD11b/CD18 (CR3, Mac-1), CD11c/CD18 (CR4, p150/95) and CD11d/CD18. The role of LFA-1 in lymphocyte trafficking is well characterised, however, the role of CD11d/CD18 is still unexplored [21]. Although the function of CD11b/CD18 and CD11c/CD18 is being investigated for long, dissecting their individual role is technically challenging for many reasons. It is important to emphasize that their expression pattern is fundamentally different in mice and men. In the mouse CD11b is expressed on all myeloid cells, while CD11c is present mainly on dendritic cells. Due to this differential expression in mice signal transduction via CD11b/CD18 is well characterized [13, 15] but is not fully known in the case of the other β2 integrins. Activation of β2 integrins was shown to be linked to Src family kinases Hck and Fgr further leading to Syk signalling via DAP12, the adaptor molecule in murine neutrophils and macrophages [13, 14, 22].

In contrast to mice, in humans CD11b and CD11c are simultaneously expressed on a wide variety of myeloid cells, as well as on certain populations of lymphoid cells [8, 23, 24]. What makes their analysis even more challenging is their overlapping ligand specificity. They have several common ligands, including inactivated C3b fragment (iC3b), fibrinogen and ICAM-1 [6, 10, 11, 25–27], and due to this it has been postulated that the function of CR3 and CR4 is similar. Namely, they mediate adhesion to ICAM-1 and fibrinogen and phagocytosis of iC3b opsonised particles. However, from an evolutionary point of view it does not seem economical to express two different receptors with identical functions by the same cell. Moreover, the intracellular domain of CD11b and CD11c in humans differ in length and amino acid sequence [1, 24, 28], which suggests functional differences between CR3 and CR4. Our goal was to dissect the functions of CR3 and CR4 in the human system.

Previously we analysed the iC3b mediated phagocytosis of human MDDCs and concluded that CR4 does not take part in this process [17]. In the present work we focused our attention on adhesion, the other main function of β2 integrins. The importance of this integrin mediated function is clearly seen in the pathologic condition of lymphocyte adhesion deficiency (LAD) syndromes type I, II and III, where defective adhesive properties of leukocytes lead to recurrent and severe life threatening infections [3]. To explore the differences between CR3 and CR4 we tested the adhesive capacity of human monocytes, MDDCs and MDMs on fibrinogen coated surface.

Fibrinogen has medium affinity to both CD11b and CD11c, suggesting that this ligand binds to both receptors, which might compete for the ligand. Examination of the affinity of fibrinogen binding to a 200 amino acid long recombinant CD11b I domain revealed in one study a K_d_ of 2,2x10^-7^M [11], while others have shown an affinity of K_d_ = 2x10^-4^M [29]. In the case of CD11c a K_d_ = 5x10^-5^M was determined for fibrinogen [29]. It has also been shown that both CD11b and CD11c can bind the large variant of fibrinogen (fibrinogen-420), further supporting the idea of simultaneous and competitive binding [7].

Here, using different techniques we show, that CD11c/CD18 is the main receptor that mediates strong adhesion of MDMs and MDDCs to fibrinogen. We have to keep in mind that the various methods used in the study reveal different aspects of adhesion. Namely, the static end-point adhesion assay measures the amount of cells capable of adhering to the substrate, while the computer controlled micropipette assay measures the strength of adhesion of the same cells. By blocking CD11c the strength of adherence was strongly reduced in the case of all analysed cell types, and the contact area of MDMs was significantly smaller than that of the other cell types. Surprisingly, blocking CD11b results in an even stronger adhesion of MDMs and MDDCs, along with a larger and more polarized contact area. These data suggest that although CD11b/CD18 is able to bind fibrinogen [10, 25], it can have a negative role in the adhesion of these two cell types. Nevertheless, in the case of monocytes, blocking both CD11b and CD11c decreased the force of adhesion. To resolve this paradoxon, we propose the following hypothesis. Adhesion to fibrinogen is dependent on the total number of CD11b/CD18 and CD11c/CD18 receptors on the cell surface. In a preliminary experiment we found that the number of fibrinogen ligands on the adhesive surface is comparable with the total amount of receptors found on monocytes. Since there is enough ligand available for both receptors, this suggests that in the case of monocytes, there is no competition between the two receptors for ligand binding, rather both take part in the adhesion process equally. This idea needs further support by analysing the CD11b/CD11c mediated adhesion of neutrophils, which express the two receptors in similar amounts to monocytes. In previous adhesion studies performed on monocytes and neutrophil granulocytes, both CD11b and CD11c mediated adhesion and spreading, suggesting that the adhesion properties of these cell types are similar [30–34]. However, MDMs and MDDCs bear far more receptors than the number of accessible ligands on the surfaces we constructed, thereby CD11b/CD18 and CD11c/CD18 compete for ligand binding. Our hypothesis raises further questions about cell adhesion under inflammatory conditions, when the number of ligands is increased and the amount of receptors also changes [8, 35]. Furthermore the expression and role of CD11d/CD18 in binding to physiological fibrinogen would be worth to study in more detail, since this receptor was also suggested to be able to bind this ligand [36].

Adhesion properties of myeloid cells, especially monocytes and neutrophils, are of particular importance in several pathologic conditions. They play an important role in atherosclerosis, where fibrinogen accumulation is detected under the endothelial layer, furthermore, monocytes were shown to upregulate CD11b and CD11c expression under hypertriglyceridemic conditions [37, 38] or in rheumatoid arthritis, where elevated CD11b levels and enhanced adhesive properties of monocytes were already shown [39]. Another pathophysiologic aspect might be related to the rs1143679 (R77H) SNP of the *ITGAM* (CD11b) gene, that is associated with systemic lupus erythematosus [40]. In this case impaired function of monocytes and MDMs, MDDCs and neutrophil granulocytes of the risk allele carrying patients was shown [41–44]. Our results shown here suggest that the impaired function may be not only the consequence of compromised CD11b, but could also be caused by the enhanced functionality and ligand binding capacity of CD11c in the absence of its competitor. This might affect mainly the functions of macrophages and dendritic cells, and thereby the adaptive immune response generated by these antigen-presenting cells. The importance of cell adhesion under pathologic conditions is further highlighted by the role of β2 integrins in forming physiological podosomes and invadosomes of cancer cells [45–48]. These structures mediate the invasion and migration of transformed cells by ECM degradation. Moreover it was shown that proteolytic digestion of fibrinogen, a component of the ECM, enhances its recognition by CD11c on human neutrophils [29].

Our recent findings highlighting the difference between the function of human CD11b/CD18 and CD11c/CD18 facilitates an even more detailed analysis of the individual role of these molecules. The state-of-the-art biophysical methods we used provide a yet unexploited potential for the analysis of cell functions, like adhesion under steady state and pathological conditions [19, 20, 49]. The possibility of cell type specific competition or cooperation between CR3 and CR4 raises several questions regarding integrin functions however the role of additional receptors in adhesion to fibrinogen cannot be excluded. Still, our results contribute to a better understanding of the distinct functions of CR3 and CR4. Whether the number and the type of the ligands they bind, or the ratio of the receptors expressed will determine the outcome of the interaction, needs further investigation.

## Materials and Methods

### Ethics statement

The study was conducted in accordance with the ethical guidelines of Declaration of Helsinki and approved by the Hungarian Medical Research Council Scientific and Research Committee (ETT TUKEB, permission number: 55627/2012/EKU). Blood samples were purchased from the Hungarian Blood Transfusion Service, where an informed written consent was obtained from all the donors.

### Isolation of monocytes

Monocytes were isolated from buffy coat obtained from healthy donors and provided by the Hungarian National Blood Transfusion Service. Peripheral blood mononuclear cells (PBMC) were separated by Ficoll-Paque PLUS (GE Healthcare Life Sciences) density gradient centrifugation and monocytes were isolated negatively by using the Miltenyi Monocyte Isolation Kit II.

### Generation of monocyte-derived macrophages (MDMs) and monocyte-derived dendritic cells (MDDCs)

To generate MDMs and MDDCs monocytes were isolated by Miltenyi CD14 MicroBeads to obtain high yield of cells. The isolated cells were cultivated for 5 days in RPMI-1640 medium (Sigma-Aldrich) supplemented with 10%FCS (Sigma-Aldrich), and Gentamicin antibiotics (Sigma-Aldrich). To generate MDMs 100 ng/mL rHu GM-CSF (R&D systems) was added to the isolated monocytes. To generate MDDCs 100 ng/mL rHu GM-CSF (R&D systems) and 15 ng/mL rHu IL-4 (R&D systems) were added to the monocytes [50–52]. Cytokines were supplied on day 3 of differentiation. To identify differentiated MDMs and MDDCs at day5 of cultivation we checked the cultures by flowcytomery: dendritic cells are CD14-, while macrophages are CD14+. Furthermore, we analyse the cultures by inverted microscope. Dendritic cells are non-adherent at day5 with several dendrites in contrast to macrophages, which are attached to the culture plate, and have a rounded shape without dendrites. The populations were found to be 95%< homogenous in size, granulation and differentiation stage as determined by flow cytometric measurements.

### Determination of absolute receptor numbers on cell surface

Absolute numbers of CD11b and CD11c was determined on the surface of monocytes, MDMs and MDDCs by using Qifikit (Dako) according to the manufacturers’ instructions. Briefly, cells were incubated with unlabelled mouse monoclonal antibodies specific for either CD11b or CD11c at saturating concentrations. After that cells were labelled with goat-anti-mouse FITC secondary antibody. A calibration curve was determined using beads that carry defined amount of mouse IgG to specify the correlation between fluorescence intensity and number of antibodies bound. This equation was used to determine the number of bound anti-CD11b or anti-CD11c antibodies on the cells’ surface. The same unlabelled mouse monoclonal antibodies were used as for receptor blocking in saturating concentration that was previously titrated by flow cytometry.

### Analysis of integrin conformational state

Integrins’ ligand binding properties highly depend on their conformation. To this end we incubated the cells with monoclonal antibody mAb24 (Hycult Biotech) that recognizes the high affinity conformation of CD18. After that, cell-bound mAb24 was labelled with goat-anti-mouse IgG Alexa488 (Molecular Probes, Invitrogen). Samples were analysed on BD FACS Calibur flowcytometer using CellQuest software for data acquisition and FCS Express 3.0 software for data analysis. We compared the cells mAb24 staining in the media used for adhesion at 4°C and 37°C compared to isotype control staining in each case.

### Blocking of CD11b/CD18 and CD11c/CD18 by antibodies

The role of CD11b/CD18 and CD11c/CD18 in the adhesion to fibrinogen was analysed by comparing the adhesive properties of monocytes, MDMs and MDDCs treated with either anti-CD11b antibody (monoclonal mIgG1 clone TMG6-5, provided by István Andó at BRC Szeged, Hungary) or anti-CD11c antibody (monoclonal mIgG1 clone 3.9, Biolegend). Both antibodies are specific for the ligand binding domain of the integrins and were used in sterile, azide-free form at saturating concentration previously titrated by flow cytometry. Cells were incubated with the receptor-specific antibodies for 30min at 4°C and used in adhesion studies without washing. Since unoccupied integrins are known to recycle to the cell surface, and would decrease the efficiency of blocking, unbound antibodies were not washed away. Cells were incubated in the presence of FcR blocking reagent (Miltenyi Biotech), and the effect of receptor specific antibodies was compared to untreated samples that were incubated only with FcR blocking reagent.

### RNA silencing in macrophages

RNA silencing was performed according to the method of Prechtel [53]. We used commercially available predesigned Qiagen (Germany) AllStar Negative control siRNA and Qiagen Genome Wide predesigned siRNA for CD11c (Hs_ITGAX_6) and CD11b (Hs_ITGAM_5). Cells were transfected on day3 and day5 of differentiation with 20μg siRNA to generate CD11c silenced, CD11b silenced or negative control silenced MDMs at day6. The expression of CD11c and CD11b was analysed on day6 by cytofluorimetry and subsequent experiments were carried out on the same day.

### Analysis of adhesion by confocal microscopy

Wells were coated by 10μg/ml fibrinogen in phosphate buffered saline solution (PBS) for 1 hour at 37°C. After that wells were washed 2 times with PBS and free surfaces were blocked with synthetic copolymer poly(L-lysine)-*graft*-poly(ethylene glycol) (PLL-*g*-PEG, SuSoS AG) for 30min at RT. After washing 2 times with PBS, 5x10^4^ cells in RPMI1640-10%FCS were immediately transferred to the wells and let to adhere for 30min at 37°C in a CO_2_ incubator. After the incubation samples were fixed by 2% paraformaldehyde (Sigma-Aldrich) for 10min and unbound cells were removed by extensive washing 2 times with PBS.

The number of adhered cells was determined by staining the nuclei with Draq5 (BioLegend) diluted 2000x in PBS and incubated for 15min at RT. Samples were analysed by Olympus IX81 confocal microscope (10x objective) and FluoView500 software. 4 representative fields were scanned in each well of triplicate sample, thereby the number of adhered cells was determined in 12 parallel fields for each treatment by ImageJ software.

To analyse the contact zone of the cells actin cytoskeleton was stained with phalloidin-Alexa488 (Molecular Probes, Invitrogen). The probe was 100x diluted in PBS-0,1% Triton-X (Sigma-Aldrich) and cells were stained for 5min at 37°C and after that washed 3 times with PBS. Samples were analysed by Olympus IX81 confocal microscope (60x objective) and FluoView500 software. Pictures were further analysed by ImageJ software.

### Analysis of adhesion with the computer-controlled micropipette

Single cell adhesion force was analysed with an imaging-based automated micropipette (CellSorter) as described previously [49, 54]. Briefly, Petri dishes were coated by 10μg/ml fibrinogen in phosphate buffered saline solution (PBS) for 1 hour at 37°C. Dishes were washed 2 times with PBS and the surface was blocked with the synthetic copolymer poly(L-lysine)-graft-poly(ethylene glycol) (PLL-g-PEG, SuSoS AG) in order to inhibit non-specific cell adhesion for 30 min at RT. After washing the Petri dish again with PBS, 7,5x10^4^ cells in RMPI-10% FCS were placed onto the coated surface. Cells were incubated for 30 minutes at 37°C in 5% CO2 atmosphere. Cultures were washed 3–4 times with Hanks’ Balanced Salt solution with sodium bicarbonate without phenol red buffer (HBSS, Sigma) to remove floating cells. Region of interest (ROI) of the Petri dish was scanned by a motorized microscope (Zeiss Axio Observer A1) equipped with a digital camera (Qimaging Retiga 1300 cooled CCD). Cells were automatically recognized by the CellSorter software. To minimize the duration of the measurement, the shortest path of the micropipette was calculated by software [55]. Individual cells were visited and probed by the glass micropipette. Micropipette with an aperture of 70 μm approached the surface to a distance of 10 μm. Vacuum was generated in a standard syringe connected to the micropipette via a high speed normally closed fluid valve. To probe cell adhesion the valve was opened for 20 ms generating a precisely controlled fluid flow and corresponding hydrodynamic lifting force acting only on the targeting cell. The hydrodynamic lifting force was calculated by running computer simulation solving the Navier-Stokes equation in a geometry corresponding to the experimental setup [49]. After each cycle of the adhesion force measurement the ROI of the Petri dish was scanned again, and the vacuum was increased to the next level. The micropipette visited again each location determined after the initial scan. Suction force was increased as long as most of the cells were removed. We counted the number of cells in the images before and after each cycle of the adhesion force measurement and calculated the ratio of still adhering cells of the population placed onto the surface at the beginning of the experiment.

### Analysis of adhesion by EPIC BT biosensor measurement

Kinetic of the adhesion was measured on the Corning EPIC biosensor as described previously in details [20]. Briefly, each well of a standard microtiter plate contains an optical grating at its bottom which permits the illuminating light to be incoupled in the waveguide. Light beams in the waveguide interfere with each other; destructive interference precludes wave guiding, while constructive interference leads to resonance and to the excitation of a guided light mode. The latter can be achieved only at a discrete illuminating wavelength, called resonant wavelength (λ). The guided light mode generates an exponentially decaying evanescent field in a 100–200 nm thick layer over the sensor, which probes the local refractive index (RI) at this interface. Any process accompanied by RI-variations in this layer (bulk RI change, molecular adsorption, cell spreading, or dynamic redistribution in the cells) untunes the resonance by altering the phase-shift of the propagating light when it is reflected from the interface (leading to destructive interference at the original resonance wavelength). The primary output of the EPIC sensor is then the shift of the resonant wavelength, Δλ. Wells were coated by 10μg/ml fibrinogen in phosphate buffered saline for 1 hour at 37°C. After that wells were washed 3 times with PBS and free surfaces were blocked with synthetic copolymer poly(L-lysine)-graft-poly(ethylene glycol) (PLL-g-PEG, SuSoS AG) for 30min at RT. After washing 3 times with PBS, 2x10^4^ cells in RPMI-10%FCS were immediately transferred to the wells and the registration of the Δλ was continuously monitored throughout the experiment (120min).

### Statistical analysis

Two-way ANOVA with Bonferroni post-test or paired t-test was used to determine significant differences between the differently treated groups, p<0,05 was considered significant. In each case a minimum of 3 independent donor’s data were analysed.
